# Drug Repurposing for Candidate SARS-CoV-2 Main Protease Inhibitors by a Novel *In Silico* Method

**DOI:** 10.3390/molecules25173830

**Published:** 2020-08-23

**Authors:** Milan Sencanski, Vladimir Perovic, Snezana B. Pajovic, Miroslav Adzic, Slobodan Paessler, Sanja Glisic

**Affiliations:** 1 Laboratory of Bioinformatics and Computational Chemistry, Institute of Nuclear Sciences Vinca, National Institute of the Republic of Serbia, University of Belgrade, 11001 Belgrade, Serbia; sencanski@vinca.rs (M.S.); vladaper@vinca.rs (V.P.); 2 Department of Molecular Biology and Endocrinology, VINCA Institute of Nuclear Sciences, National Institute of the Republic of Serbia, University of Belgrade, 11001 Belgrade, Serbia; pajovic@vinca.rs (S.B.P.); miraz@vin.bg.ac.rs (M.A.); 3 Department of Pathology, University of Texas Medical Branch, Galveston, TX 77555, USA; slpaessl@utmb.edu; 4 Institute for Human Infections and Immunity, University of Texas Medical Branch, Galveston, TX 77555, USA

**Keywords:** SARS-CoV-2, main protease M^pro^, drug repurposing, virtual screening, ISM

## Abstract

The SARS-CoV-2 outbreak caused an unprecedented global public health threat, having a high transmission rate with currently no drugs or vaccines approved. An alternative powerful additional approach to counteract COVID-19 is *in silico* drug repurposing. The SARS-CoV-2 main protease is essential for viral replication and an attractive drug target. In this study, we used the virtual screening protocol with both long-range and short-range interactions to select candidate SARS-CoV-2 main protease inhibitors. First, the Informational spectrum method applied for small molecules was used for searching the Drugbank database and further followed by molecular docking. After *in silico* screening of drug space, we identified 57 drugs as potential SARS-CoV-2 main protease inhibitors that we propose for further experimental testing.

## 1. Introduction

An outbreak of the novel coronavirus disease (COVID-19) in December of 2019 in Wuhan, China, has spread promptly to more than 213 countries, with over 1,918,138 confirmed cases and over 123,126 confirmed deaths worldwide as of 15 April 2020 [1]. The outbreak has been declared a global pandemic by The World Health Organization (WHO) on 11 March 2020 [1]. It is uncertain whether a COVID-19 pandemic will cause multiple concurrent epidemics over one to three years, and SARS-CoV-2 may become an endemic virus globally [2]. Moreover, millions of people have been disturbed as a result of mandatory isolations/quarantines and every part of society is severely affected, with health care systems and economy adversely affected [3].

The outbreak caused by SARS-CoV-2 is an unprecedented global public health threat owing to the high transmission rate of the virus, coupled with currently no drugs or vaccines approved. In the pandemic setting with rapid virus transmission, new vaccine production is of exceptional importance, besides much needed therapeutic, that are both expected to need months to years to develop. The rapid response action to the emergent pandemic is repurposing of approved antiviral, antimalarial, antiparasitic agents and those based on immunotherapy approaches to treat COVID-19, with some clinical trials already started [4,5,6].

An alternative efficient additional strategy to tackle COVID-19 is *in silico* drug repurposing approaches. The main protease M^pro^, also called 3CLpro, represents an attractive drug target due to its essential role in the viral life cycle, crucial for viral replication. The pp1a and pp1ab, two overlapping polyproteins, important for viral replication and transcription, are encoded by the SARS-CoV-2 replicase gene [7,8]. The M^pro^ cleaves large polyprotein 1ab in at least 11 sites. The M^pro^ is highly conserved across the Coronaviridae family and any mutation here can be disastrous for the virus [9,10]. As one of the best-characterized drug targets among coronaviruses, in the absence of closely related human homologues, the M^pro^ represents one of the most attractive SARS-CoV-2 drug targets. Since there is no human protease with similar cleavage specificity, the inhibitors are expected to be nontoxic [11]. SARS-CoV-2 M^pro^ is active in a dimer form, consisting of two monomers arranged nearly perpendicular to one another [11]. The dimerization is necessary for the M^pro^ enzymatic activity as the N-finger of each of the two monomers interacts with Glu166 of the other monomer support the correct orientation of the S1 pocket of the substrate binding site. M^pro^ active site comprises a catalytic dyad that consists of the conserved residues H41 and C145 [9]. The available high-resolution experimental structure of the main protease of SARS-CoV-2 was used in the current study as the target for molecular docking-based virtual screening (VS) [7].

In this study, we used VS protocol with sequential filters, based on the both long-range and short-range interactions, to select candidate SARS-CoV-2 M^pro^ inhibitors. First, the Informational spectrum method applied for Small Molecules (ISM-SM) was used for searching Drugbank database [12], and further was followed by molecular docking. By applying a new combo filter, we select 57 compounds for further experimental testing. The use of such protocol is of great importance in case of drug repurposing, for it can precisely determine protein domains where the possible binding site is placed, and select small molecules that could specifically bind to those domains. In addition, due to the simplicity of ISM-SM, a large number of compounds can be rapidly screened with little effort in data preparation. In particular, due to COVID-19 fast expansion, a VS protocol that could bring promising new drug candidates is of great importance.

## 2. Results

### 2.1. Informational Spectrum Method Analysis

**Figure 1:**
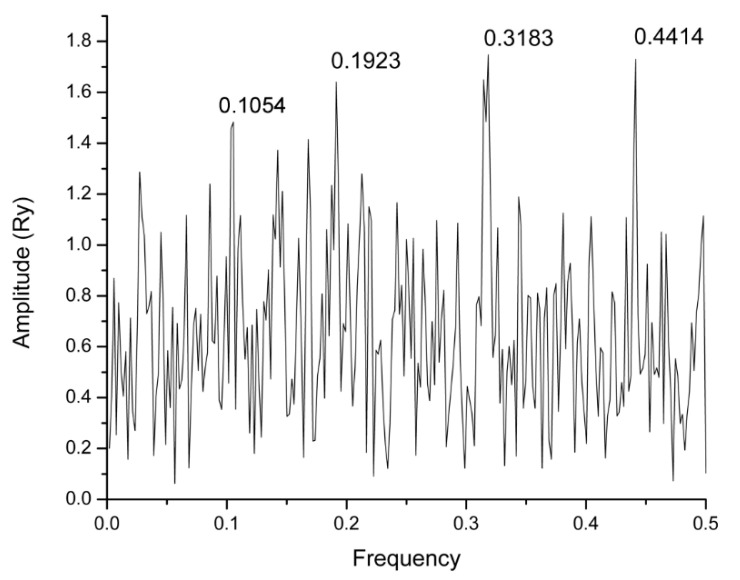
Informational spectrum (IS) of SARS-CoV-2 M^pro^.

**Figure 2:**
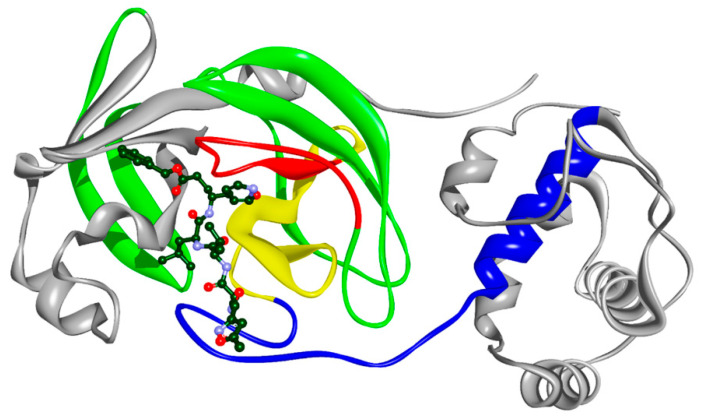
Crystal structure of M^pro^. Marked regions correspond to F(0.1923)—red (residues 131–195), F(0.3183)—yellow (residues 151–183), F(0.4414)—green (residues 72–136) and F(0.1054)—blue (residues 182–214). Note that regions F(0.1923) and F(0.3183) overlap, as well as F(0.4414) and F(0.1923); also F(0.3183) and F(0.1054). The bound compound is the co-crystalized inhibitor N3.

**Figure 3:**
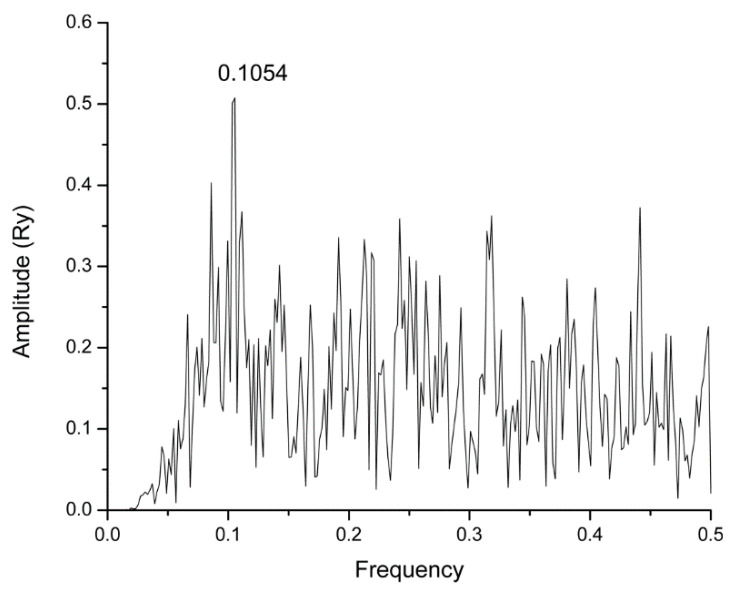
Cross-spectrum (CS) of M^pro^ and Ebselen.

In the present study, we have used the Informational spectrum method (ISM) for the structure/function analysis of the highly conserved SARS-CoV-2 protein M^pro^. According to the previous studies, the informational characteristic of the protein, identified in the analysis, corresponds to the protein key biological function. The informational spectrum (IS) of M^pro^ contains three characteristic peaks at the frequencies F(0.1923), F(0.3183) and F(0.4414), shown in Figure 1. To find the domains of a protein crucial for the information related to the three frequencies, M^pro^ was computationally scanned. As a result of scanning with the ISM algorithm, with overlapping windows of different lengths, we identified regions with the highest amplitudes at these frequencies. It was shown that the regions, including residues 131–195, 151–183 and 72–136, are essential for the information represented by the frequency F(0.1923), F(0.3183) and F(0.4414), respectively. Two dominant frequencies of M^pro^, F(0.1923) and F(0.3183), correspond to the catalytic domain of the enzyme, while F(0.4414) to the allosteric domain (Figure 2). In the recent study, Ebselen has shown *in-vitro* M^pro^ inhibition activity [7]. We calculated cross-spectrum (CS) for M^pro^ and Ebselen and found a dominant peak at the F (0.1054) (Figure 3). Due to the importance, we additionally marked this frequency among three others (Figure 1). This frequency was computationally mapped to domain 182–214, corresponding to the allosteric domain. We further searched CS of Drugbank [12] candidates, with M^pro^ at the F(0.1923), F(0.3183), F(0.4414) and F (0.1054), to find potential M^pro^ inhibitor candidates, with additional condition that candidates’ IS contained main peaks on those values. With this search, we selected 57 candidate drugs (Table 1 and Table 2).

### 2.2. Molecular Docking

For further filtering of the selected compounds, we carried molecular docking into the catalytic and allosteric domains of SARS-CoV-2 (Table 1 and Table 2). The cut-off binding energy value for the best candidates was set to −7.0 kcal/mol. From the initial docking, the best candidates were found to be mezlocillin, camazepam and spirapril, targeting the catalytic site. Raltegravir, rolitetracycline, tolvaptan, ciclesonide and rescinnamine were found targeting the allosteric domain. All compounds have a better docking score than ebselen, which suggests that they could be potentially promising inhibitors of SARS-CoV-2 M^pro^. Because ebselen is an inhibitor of HIV-1 capsid C-terminal domain dimerization [13], from this study is assumed that it analogously hinders M^pro^ dimerization.

**Figure 4:**
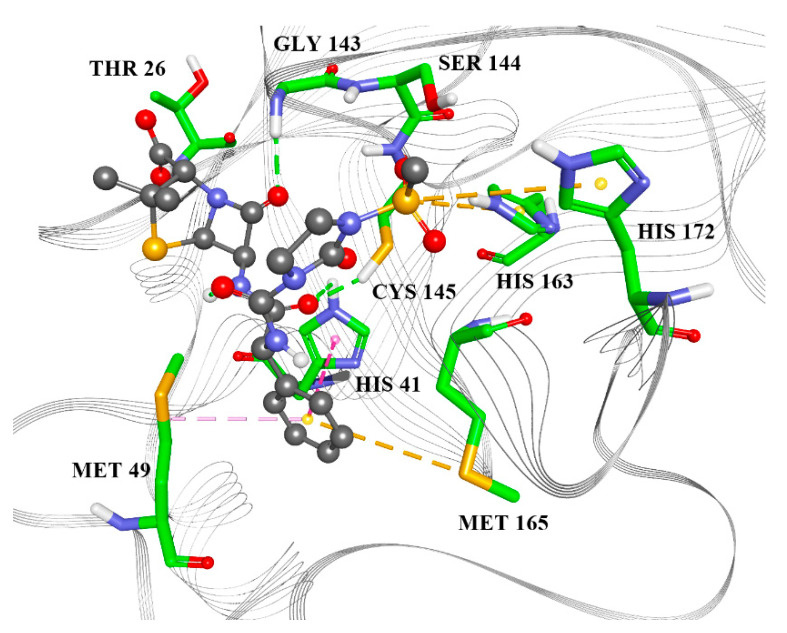
Mezlocillin in the M^pro^ catalytic site. Green lines—hydrogen bonds; purple—alkyl-π interactions, magenta—π–π interactions, yellow—S–π interactions.

Docking in the catalytic site showed that all candidates (Table 3, Figure 4, Figures S1–S3 in the Supplementary Materials) interact with Cys 145 and His 41, which are essential for the catalytic activity of M^pro^ [11]. Types of intermolecular interactions that candidates form with amino acid residues are hydrogen bonds, aromatic π–π, alkyl–π, S–π and cation–π interactions.

**Figure 5:**
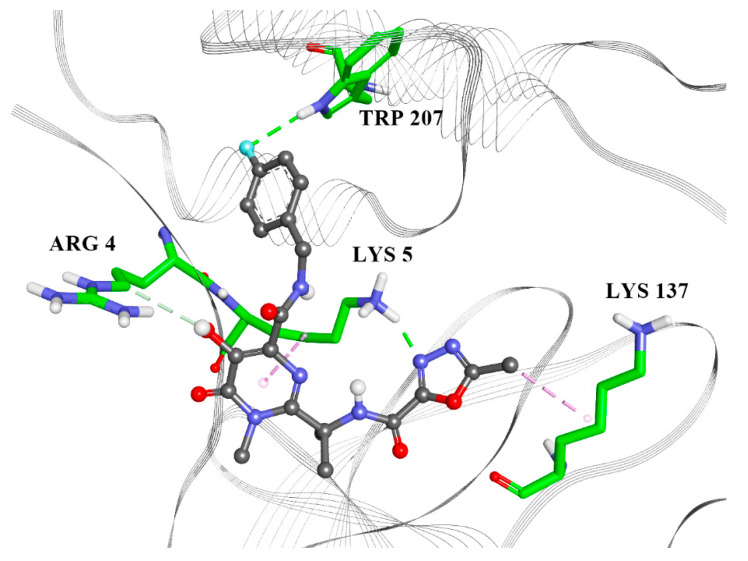
Raltegravir in the M^pro^ allosteric site. Green lines—hydrogen bonds; purple—alkyl–π/hydrophobic interactions; white line—carbon hydrogen bond.

From the docking results in the allosteric site (Table 4, Figure 5, Figures S4–S7 in the Supplementary Materials), noticeable protein–ligand interactions are with Lys 5, which is next to Arg 4, an essential residue for the dimerization process [11]. The other interacting residues (Lys 137, Gly 138, Glu 290, Tyr 126 and Leu 286) are in accordance with those found from the biological assembly in PDB 6LU7 (Table S1 in the Supplementary Materials).

In addition, by calculating CS of the drug, its main target and M^pro^, we give a proof of the concept of our method, implicating that the drug targets both proteins. For instance, in Figure S9 is presented CS of mezlocillin, M^pro^, and d-alanyl-d-alanine carboxypeptidase (Uniprot code Q75Y35), one of its main targets. For all three entities, the common frequency value in CS is at F(0.1923). CS of docking top hits, M^pro^, and their main targets are presented in Figures S8–S14 (in the Supplementary Materials).

## 3. Discussion

Current prevention and treatment options for SARS-CoV-2 infections are insufficient due to lack of approved drug therapy or vaccines [10]. In a search for preventive and therapeutic options to counter threats of pandemics, the fundamental problem is that drug development is a costly, time-consuming and risky enterprise. Therefore, drug repurposing is a promising therapeutic strategy for many viral diseases and the most realistic in the present pandemic. Various predictive *in silico* approaches have been applied to identify drug repositioning opportunities against SARS-CoV-2 [10].

The manuscript’s originality lies in applying an innovative concept in selecting candidate molecules for the treatment of SARS-CoV-2 infection, which includes molecular characteristics responsible for long-range recognition and targeting between interacting biological molecules. The VS protocol applied in this study is based on combined *in silico* approaches considering both short- and long-range interactions between interacting molecules.

In this work, we have used the ISM for the structure/function analysis of the highly conserved SARS-CoV-2 protein M^pro^ and identified the key informational characteristic of the protein, which corresponds to the protein key biological function. The ISM was recently used for prediction of potential receptor, natural reservoir, tropism, and therapeutic/vaccine target of COVID-19 [14]. In another recent study, ISM was used for the analysis of the COVID-19 Orf3b, suggesting that this protein acts as a modulator of the interferon signaling network [15]. To select drug candidates for SARS-CoV-2 M^pro^ inhibitor, further in this work, we used the VS protocol based on the application of successive filters. First, the ISM-SM was used for the fast screening of large compound libraries through candidate selection at a specific frequency value. Molecules were treated as quasi-linear entities, analogous to peptides, and ISM was applied to predict potential candidates for the SARS-CoV-2 M^pro^. Previously this novel approach in bioinformatics treatment of small molecules was successfully used for analyzing GPCR drugs of the Golden dataset [16] with amino acid sequences of corresponding receptors. The essential information that can be extracted from protein–ligand CS spectra is the domain of the binding site in the corresponding receptor [17]. As the second step of VS to meet short-range compatibility, we used molecular docking.

The same drugs already selected in other studies as SARS-CoV-2 M^pro^ inhibitors represent proof-of-concept for our novel *in silico* method. In this manuscript, several new SARS-CoV-2 M^pro^ candidate inhibitors were also proposed. One of the best ranked allosteric inhibitors from our computational study is ciclesonide. Another computational study also found ciclesonide as a potential inhibitor of M^pro^ [18]. In *in-vitro* studies, ciclesonide showed good antiviral activity against SARS-CoV-2, however against a different target [19]. The potential multitarget activity of ciclesonide may help to overcome drug resistance in COVID-19. Additional favorable results were reported from studies identifying that ciclesonide inhalant may improve the respiratory status in severe COVID-19-induced pneumonia [20] and in cases of mild- to mid-stage COVID-19 [21]. Ciclesonide is a safe drug commonly used for inhalation in premature babies and newborns, as well as the elderly. It is effective in controlling chronic inflammation of the respiratory tract and the only steroid that showed anti-SARS-CoV-2 activity [21]. These studies gave rise to a recently initiated an open-labeled, randomized Phase 2 clinical trial to evaluate the antiviral effect of ciclesonide on the reduction of viral load in patients with mild COVID-19 [22].

Raltegravir, the first approved human immunodeficiency virus type 1 (HIV-1) integrase inhibitor and the best M^pro^ allosteric inhibitor according to our study, was among the 30 compounds, with potential SARS-CoV-2 activity shown by a joint research team of the Shanghai Institute of Materia Medica and Shanghai Tech University against SARS-CoV-2 from *in silico* and *in-vitro* analysis [23]. Another of the best ranked allosteric inhibitors from our computational study was rolitetracycline, the first of the semi-synthetic tetracyclines. In recent molecular docking study it also showed the best binding with the catalytic center of the SARS-CoV-2 M^pro^ through binding with CYS 145 and HIS 41 [24]. Tolvaptan is also in the group of potentially the best M^pro^ allosteric inhibitors. The efficacy and safety of tolvaptan therapy was reported in patients with the COVID-19-associated syndrome of inappropriate antidiuretic hormone secretion [25,26].

In our work, mezlocillin was the best candidate for catalytic site inhibitor of M^pro^. The same result was also reported from another *in silico* study [27]. The second best candidate for catalytic site inhibitor, camazepam, is a benzodiazepine. Although an anxiolytic, there have been earlier reported antiviral activities of benzodiazepines [28]. According to our study, spirapril and ACE inhibitors could be a promising catalytic site inhibitor candidate of SARS-CoV-2 M^pro^. It was proposed that ACE inhibitors could have both potentially harmful and beneficial effects on COVID-19. Membrane-bound angiotensin-converting enzyme 2 (ACE2) participates in the entry of SARS-CoV-2 into human cells, and animal studies show that ACE inhibitors could upregulate ACE2 expression; and on the other side, the beneficial effect can be expected with upregulated ACE2 converting angiotensin II to angiotensin-(1–7), with potentially advantageous vasodilatory and anti-inflammatory properties [29,30]. We can assume that ACE inhibitors through M^pro^ inhibition, in addition to the already assumed theory of the advantageous effect of upregulated ACE2, could explain that treatment with ACE-inhibitors is associated with less severe disease in SARS-CoV-2 infection [31].

A number of drugs selected in our study as repositioning candidates that potentially bind to the catalytic site were also identified in other *in silico* studies that analyzed the same target. In the molecular docking study, bacampicillin was among the best repurposed drugs against the main protease of SARS-CoV-2 [32]. Carbinoxamine showed in another docking study potential activity against SARS-CoV-2 M^pro^ [33]. In the docking study of FDA approved drugs against protease and spike protein of COVID-19, paromomycin was found to have a strong binding affinity against both Spike and M^pro^ of SARS-CoV-2 according to its glide score [34]. Phensuximide was found in molecular docking simulations of FDA-approved small compounds associated with protection against COVID-19, M^pro^ [35]. In VS-based study, magnesium ascorbate, a buffered (non-acidic) form of vitamin C, was found to be the top lead compound among 106 nutraceuticals against SARS-CoV-2 s M^pro^ [36], and tizanidine was amongst the 11 approved drugs predicted to show a high binding affinity in VS study with M^pro^ [37].

The potential allosteric inhibitors found in our study were also selected as M^pro^ inhibitors in several *in silico* studies. In the VS study, cefotiam was found among eight compounds potential SARS-CoV-2 M^pro^ inhibitors [38]. In another docking study, voriconazole, tobramycin and kanamycin showed potential activity against SARS-CoV-2 M^pro^ [33,37]. Ospemifene was among 51 hits selected *in silico* against M^pro^ [39], and propylthiouracil was among the top 20 drugs showing the highest docking score [40]. Besides, we found oseltamivir, the influenza neuraminidase inhibitor, to have a higher docking score against SARS-CoV-2 M^pro^ than ebselen (Table 2). The use of oseltamivir was already reported during the COVID-19 epidemic in China, either with or without antibiotics, and clinical trials are ongoing with oseltamivir with multiple combinations with chloroquine and favipiravir [41,42].

The benefit of the ISM-SM approach over other *in silico* approaches lies in its crucial ability to determine the long-range molecular recognition between protein and ligand. Due to low demanding data preparation, requiring only protein sequence and SMILES molecules notation of drug candidates, it enables quick scanning of large molecular libraries.

The use of structural models of protein–drug complexes, combined with the ISM-SM approach, can improve inhibitors against M^pro^. Novel or modified ligands should maintain ISM spectrum features of the known inhibitors by targeting a specific domain in M^pro^ (i.e., containing reported frequency values in their CS). The next step, involving short-range interaction approaches, should be oriented not only towards modifications that strengthen interactions with amino acid residues of the binding site. The introduction of functional groups that interact with near residues, identified in specific domains by ISM, should also be considered.

Selected drugs from our computational study may represent an initial step for further experimental investigations in a quest for safe, new treatments for COVID-19. The activity of inhibitors that we propose should be further experimentally confirmed.

## 4. Materials and Methods

### 4.1. Informational Spectrum Method

In this work, we analyze the SARS-CoV-2 M^pro^ protein using the informational spectrum method (ISM). A comprehensive explanation of the sequence analysis based on ISM is available elsewhere [43]. According to this approach, sequence (protein or DNA) is transformed into a signal by assignment of numerical values of each element (amino acid or nucleotide). These values correspond to electron–ion interaction potential (EIIP) [44], determining the electronic properties of amino acid/nucleotides, which are essential for their intermolecular interactions. The EIIP descriptors are easily calculated using the following formulas:

(1) $$Z^{*}=\sum_{i=1}^{m}n_{i}Z_{i}/N,$$

(2) $$EIIP=0.25Z^{*}\sin\left(1.04\pi Z^{*}\right)/2\pi ,$$

where *i* is type of the chemical element, *Z* is valence of the i-th chemical element, *n* is number of the i-th chemical element atoms in the compound, *m* is number of types of chemical elements in the compound and *N* is total number of atoms.

The EIIP signal is then transformed using fast Fourier transform (FFT) into information spectrum (IS) as a representation of a sequence in the form of a series of frequencies and amplitudes:

(3) $$X\left(n\right)=\sum_{m=1}^{N}x\left(m\right)e^{-\frac{i\pi nm}{N}},\;n=1,2,\ldots ,N/2,$$

where *m* is the summation index, *x*(*m*) is the m-th member of a given numerical “signal” series (from a transformed, encoded primary protein sequence in our case), *N* is the total number of points in this series), *n* is the number of a discrete frequency (ranging from 1 on up to *N*/2) in the DFT, *X*(*n*) are the discrete Fourier transformation amplitude coefficients corresponding to each discrete frequency *n* and 2π × (*n*/*N*) is the phase angle at each given *m* in the amino−acid series of the protein in question.

However, in the case of protein analysis, the relevant information is primarily presented in energy density spectrum, which is defined as follows:

(4) $$S\left(n\right)=X\left(n\right)X^{*}\left(n\right)={\left|X\left(n\right)\right|}^{2},n=1,2,\ldots ,N/2,$$

By this, the virtual spectroscopy method is feasible to analyze protein sequences without any previous experimental data functionally. Its extension for small molecules, ISM-SM, was developed and published recently [17]. A small molecule is imported in SMILES notation and decoded by atomic groups into an array of corresponding EIIP values. Using FFT, the corresponding IS of a small molecule is computed. This spectrum is further multiplied by IS of the protein receptor to obtain a cross-spectrum (CS). Cross-spectral function is the function which determines common frequency characteristics of two signals. For discrete series it is defined as follows:

(5) $$S\left(n\right)=X\left(n\right)\times Y{\left(n\right)}^{*},\;n=1,2,\ldots ,N/2,$$

where *X*(*n*) and DFT coefficients of the series *x*(*m*), and *Y*(*n*)* are complex conjugated DFT coefficients of the series *Y*(*m*).

From common frequencies in CS, one can determine whether protein interacts with small molecules and determine the corresponding binding region in the protein.

### 4.2. Data Preparation

Protein sequences were downloaded from the UniProt database (https://www.uniprot.org/) with the following accession numbers: Gaba alpha subunit, P14867 d-alanyl-d-alanine carboxypeptidase Q75Y35, Angiotensin-converting enzyme P12821, HIV 1 Integrase Q7ZJM1, 30S ribosomal protein S9 P0A7X3, Vasopressin V2 receptor P30518. The FASTA COVID-19 M^pro^ sequence was downloaded from RCSB, PDB ID 6LU7 and the corresponding IS was calculated. A set of 1490 approved Drugbank [12] drugs with corresponding SMILES was subjected to IS and CS calculation with M^pro^. All calculations were carried out using our in-house software.

### 4.3. Molecular Docking

Molecular docking of selected candidates into the crystal structure of M^pro^ was carried out. The receptor three-dimensional structure was downloaded from RCSB, PDB ID 6LU7 [7]. All ligands, waters and ions were removed from PDB file. Two grid boxes with dimensions 24 × 24 × 24 Å were set to span all amino acid residues interacting with co-crystallized inhibitor N3 in case of the catalytic site. For the allosteric domain, it was set to span the residues interacting in the dimer interface. The (x, y, z) centers of the grid boxes were (−11.775, 13.910, 66.706) for the catalytic site and (−22.521, 1.749, 50.782) for the allosteric domain. Selected drugs from the previous step were converted from SMILES to 3D SDF and further to PDB files, and protonated at physiological pH. Geometry optimization was carried out in MOPAC 2016 [45] at PM7 level of theory [46]. Default software settings for hydrophobic and hydrophilic terms in docking search function were used. Exhaustiveness was set to 50. Molecular docking was carried out in Autodock Vina [47], implemented in VS software PyRx [48].

For intermolecular interactions identification, the following criteria were used: For hydrogen bonds, as a maximum distance between the donor (D) and a hydrogen atom (H), D · · · H, 3.4 Å was used and angle D-H-A between 90° and 180°; for a salt bridge maximum distance, D · · ·A, 4 Å was used. For π–alkyl interactions, the maximum distance between the centroid of the aromatic ring and the C-atom of the alkyl group was 4 Å and angle 45°. Regarding S–π interactions, for edge-on type, the maximum distance was 6 Å and angle 70°; for face-on, 4.5 Å and 25°. For π–π interactions, the maximum distance between centroids was 6 Å, and values of theta and gamma angles were 50° and 35° for stacked and 30° and 55° for T-shaped conformation, respectively. For π–cation interactions, the maximum distance between cation and centroid was 4 Å and angle 40°.

Figures were made in BIOVIA Discovery Studio 2017 [49], Schrodinger Maestro 11.1 [50] and Origin 9.0 software [51].

## Figures and Tables

**Table 1:** Docking scores of the compounds binding to the catalytic site.

Name	Drugbank ID	F	Binding Energy (kcal/mol)
Mezlocillin	DB00948	0.1923	−8.6
Camazepam	DB01489	0.1923	−7.5
Spirapril	DB01348	0.1923	−7.1
Bacampicillin	DB01602	0.1923	−6.8
Bacitracin	DB00626	0.3183	−6.6
Carbinoxamine	DB00748	0.3183	−6.6
Paromomycin	DB01421	0.1923	−6.5
Nifedipine	DB01115	0.1923	−6.3
Gemfibrozil	DB01241	0.1923	−5.9
Trimethaphan	DB01116	0.1923	−5.6
Phensuximide	DB00832	0.3183	−5.6
Nitrendipine	DB01054	0.1923	−5.4
Paliperidone	DB01267	0.1923	−5.4
Levetiracetam	DB01202	0.1923	−5.2
Chlorambucil	DB00291	0.1923	−5.2
Vitamin C	DB00126	0.1923	−5.1
Tizanidine	DB00697	0.1923	−5.0
Ifosfamide	DB01181	0.1923	−4.9
Aminophenazone	DB01424	0.1923	−4.6
Mecamylamine	DB00657	0.1923	−4.3
Tazarotene	DB00799	0.1923	−4.1

**Table 2:** Docking scores of the compounds binding to the allosteric site.

Name	Drugbank ID	F	Binding Energy (kcal/mol)
Raltegravir	DB06817	0.1054	−7.9
Rolitetracycline	DB01301	0.4414	−7.6
Tolvaptan	DB06212	0.1054	−7.3
Ciclesonide	DB01410	0.1054	−7.2
Rescinnamine	DB01180	0.4414	−7.2
Spectinomycin	DB00919	0.1054	−6.8
Cefotiam	DB00229	0.1054	−6.6
Azatadine	DB00719	0.1054	−6.5
Flecainide	DB01195	0.1054	−6.2
Pivmecillinam	DB01605	0.1054	−6.2
Voriconazole	DB00582	0.1054	−6.1
Ambenonium	DB01122	0.1054	−5.9
Amitriptyline	DB00321	0.1054	−5.9
Azapropazone	DB07402	0.1054	−5.8
Miconazole	DB01110	0.1054	−5.8
Clofedanol	DB04837	0.1054	−5.7
Flutamide	DB00499	0.1054	−5.7
Leflunomide	DB01097	0.1054	−5.7
Tobramycin	DB00684	0.1054	−5.7
Clevidipine	DB04920	0.1054	−5.6
Imipramine	DB00458	0.1054	−5.6
Kanamycin	DB01172	0.1054	−5.6
Ciclopirox	DB01188	0.1054	−5.5
Oseltamivir	DB00198	0.1054	−5.5
Ospemifene	DB04938	0.1054	−5.5
Trimipramine	DB00726	0.1054	−5.5
Ebselen	DB12610	0.1054	−5.3
Bepridil	DB01244	0.1054	−5.1
Ethinamate	DB01031	0.1054	−4.9
Propylthiouracil	DB00550	0.1054	−4.5
l−Arginine	DB00125	0.1054	−4.4
Isoflurophate	DB00677	0.1054	−4.2
Ethanolamine Oleate	DB06689	0.1054	−4.1
l−Carnitine	DB00583	0.1054	−4.1
l−Lysine	DB00123	0.1054	−4.0
Methoxyflurane	DB01028	0.1054	−3.6

**Table 3:** Protein–ligand interactions in the catalytic site.

Residue	Mezlocillin	Camazepam	Spirapril
THR27	X		
HIS41	X	X	X
GLY143	X		
SER144			X
CYS145	X	X	X
HIS163	X	X	
GLU166		X	X
HIS172	X		
MET49	X	X	X
MET165	X		X

**Table 4:** Protein–ligand interactions in the allosteric domain.

	Raltegravir	Rolitetracycline	Tolvaptan	Ciclesonide	Rescinnamine
ARG4	X			X	
LYS5	X	X	X	X	X
ARG131		X	X		X
LYS137	X		X		X
TRP207	X				
ASP289		X			X
LEU287		X			
GLU288		X			
GLN127					X
ASP197					X
GLY138					X
GLU290			X		
TYR126		X			X
PHE291				X	
LEU286			X		

## Supplementary Materials

The following are available online: Figure S1: Camazepam in the M^pro^ catalytic site; Figure S2: Mezlocillin in the M^pro^ catalytic site; Figure S3: Spirapril in the M^pro^ catalytic site; Figure S4: Raltegravir in the M^pro^ allosteric site; Figure S5: Rolitetracycline in the M^pro^ allosteric site; Figure S6: Tolvaptan in the M^pro^ allosteric site; Figure S7: Rescinnamine in the M^pro^ allosteric site; Figure S8: CS of M^pro^, Gaba alpha subunit 1 and Camazepam; Figure S9: CS of M^pro^, d−alanyl−d−alanine carboxypeptidase and Mezlocillin; Figure S10: CS of M^pro^, Angiotensin-converting enzyme and Spirapril; Figure S11: CS of M^pro^, Integrase and Raltegravir; Figure S12: CS of M^pro^, 30S ribosomal protein S9 and Rolitetracycline; Figure S13: CS of M^pro^, Vasopressin V2 receptor and Tolvaptan; Figure S14: CS of M^pro^, Angiotensin-converting enzyme and Rescinnamine, Table S1: List of interacting residues in M^pro^ dimer in the biological assembly PDB ID 6LU7.

## References

[1] World Health Organization Coronavirus Disease 2019. https://www.who.int/emergencies/diseases/novel-coronavirus-2019.

[2] Yamey G., Schäferhoff M., Pate M., Chawla M., Ranson K., Hatchett R., Wilder R. (2020). Funding the Development and Manufacturing of COVID-19. Vaccines.

[3] Liu C., Zhou Q., Li Y., Garner L.V., Watkins S.P., Carter L.J. (2020). Research and Development on Therapeutic Agents and Vaccines for COVID-19 and Related Human Coronavirus Diseases. ACS Cent. Sci..

[4] Tu Y.F., Chien C.S., Yarmishyn A.A., Lin Y.Y., Luo Y.H., Lin Y.T., Lai W.T., Yang D.M., Chou S.J., Yang Y.P. (2020). A Review of SARS-CoV-2 and the Ongoing Clinical Trials. Int. J. Mol. Sci..

[5] Li G., De Clercq E. (2020). Therapeutic options for the 2019 novel coronavirus (2019-nCoV). Nat. Rev. Drug Discov..

[6] Mitjà O., Clotet B. (2020). Use of antiviral drugs to reduce COVID-19 transmission. Lancet Glob. Health.

[7] Jin Z., Du X., Xu Y., Deng Y., Liu M., Zhao Y., Zhang B., Li X., Zhang L., Peng C. (2020). Structure of M^pro^ from COVID-19 virus and discovery of its inhibitors. Nature.

[8] Wu F., Zhao S., Yu B., Chen Y.M., Wang W., Song Z.G., Hu Y., Tao Z.W., Tian J.H., Pei Y.Y. (2020). A new coronavirus associated with human respiratory disease in China. Nature.

[9] Anand K., Ziebuhr J., Wadhwani P., Mesters J.R., Hilgenfeld R. (2003). Coronavirus main proteinase (3CLpro) structure: Basis for design of anti-SARS drugs. Science.

[10] Strodel B., Olubiyi O., Olagunju M., Keutmann M., Loschwitz J. (2020). High Throughput Virtual Screening to Discover Inhibitors of the Main Protease of the Coronavirus SARS-CoV-2. Preprints.

[11] Zhang L., Lin D., Sun X., Curth U., Drosten C., Sauerhering L., Becker S., Rox K., Hilgenfeldl R. (2020). Crystal structure of SARS-CoV-2 main protease provides a basis for design of iMproved a-ketoamide inhibitors. Science.

[12] Wishart D.S., Feunang Y.D., Guo A.C., Lo E.J., Marcu A., Grant J.R., Sajed T., Johnson D., Li C., Sayeeda Z. (2017). DrugBank 5.0: A major update to the DrugBank database for 2018. Nucleic Acids Res..

[13] Thenin-Houssier S., De Vera I.M., Pedro-Rosa L., Brady A., Richard A., Konnick B., Opp S., Buffone C., Fuhrmann J., Kota S. (2016). Ebselen, a small-molecule capsid inhibitor of HIV-1 replication. Antimicrob. Agents Chemother..

[14] Veljkovic V., Vergara-Alert J., Segalés J., Paessler S. (2020). Use of the informational spectrum methodology for rapid biological analysis of the novel coronavirus 2019-nCoV: Prediction of potential receptor, natural reservoir, tropism and therapeutic/vaccine target. F1000Research.

[15] Veljkovic V., Paessler S. (2020). COVID-19 Orf3b protein: The putative biological function and the therapeutic target. (Version 1) Res. Sq..

[16] Yamanishi Y., Araki M., Gutteridge A., Honda W., Kanehisa M. (2008). Prediction of drug–target interaction networks from the integration of chemical and genomic spaces. Bioinformatics.

[17] Sencanski M., Sumonja N., Perovic V., Glisic S., Veljkovic N., Veljkovic V. (2019). The Application of Information Spectrum Method on Small Molecules and Target Recognition. arXiv.

[18] Hosseini M., Chen W., Wang C. (2020). Computational Molecular Docking and Virtual Screening Revealed Promising SARS-CoV-2 Drugs (Preprint). ChemRxiv.

[19] Matsuyama S., Kawase M., Nao N., Shirato K., Ujike M., Kamitani W., Shimojima M., Fukushi S. (2020). The inhaled corticosteroid ciclesonide blocks coronavirus RNA replication by targeting viral NSP15. BioRxiv.

[20] Nakajima K., Ogawa F., Sakai K., Uchiyama M., Oyama Y., Kato H., Takeuchi I. (2020). A Case of Coronavirus Disease 2019 Treated With Ciclesonide. Mayo Clinic Proceedings.

[21] Iwabuchi K., Yoshie K., Kurakami Y., Takahashi K., Kato Y., Morishima T. (2020). Therapeutic potential of ciclesonide inahalation for COVID-19 pneumonia: Report of three cases. J. Infect. Chemother..

[22] A Trial of Ciclesonide Alone or in Combination with Hydroxychloroquine for Adults with Mild COVID-19. https://clinicaltrials.gov/ct2/show/NCT04330586.

[23] Dong L., Hu S., Gao J. (2020). Discovering drugs to treat coronavirus disease 2019 (COVID-19). Drug Discov..

[24] Aly M.O. (2020). Molecular Docking Reveals the Potential of Aliskiren, Dipyridamole, Mopidamol, Rosuvastatin, Rolitetracycline and Metamizole to Inhibit COVID-19 Virus Main Protease. ChemRxiv.

[25] Verbalis J.G., Adler S., Schrier R.W., Berl T., Zhao Q., Czerwiec F.S. (2011). Efficacy and safety of oral tolvaptan therapy in patients with the syndrome of inappropriate antidiuretic hormone secretion. Eur. J. Endocrinol..

[26] Yousaf Z., Al-Shokri S.D., Al-soub H., Mohamed M.F. (2020). COVID-19-associated SIADH: A clue in the times of pandemic!. Am. J. Physiol.-Endocrinol. Metab..

[27] Gul S., Ozcan O., Asar S., Okyar A., Baris I., Kavakli I.H. (2020). *In Silico* Identification of Widely Used and Well Tolerated Drugs That May Inhibit SARSCov-2 3C-like Protease and Viral RNA-Dependent RNA Polymerase Activities, and May Have Potential to Be Directly Used in Clinical Trials. ChemRxiv.

[28] De Lucca G.V., Otto M.J. (1992). Synthesis and anti-HIV activity of pyrrolo-[1, 2-d]-(1, 4)-benzodiazepin-6-ones. Bioorg. Med. Chem. Lett..

[29] Smyth L.J., Cañadas-Garre M., Cappa R.C., Maxwell A.P., McKnight A.J. (2019). Genetic associations between genes in the renin-angiotensin-aldosterone system and renal disease: A systematic review and meta-analysis. BMJ Open.

[30] Fang L., Karakiulakis G., Roth M. (2020). Are patients with hypertension and diabetes mellitus at increased risk for COVID-19 infection?. Lancet Respir. Med..

[31] Bean D., Kraljevic Z., Searle T., Bendayan R., Pickles A., Folarin A., Roguski L., Noor K., Shek A., O’Gallagher K. (2020). Treatment with ACE-inhibitors is associated with less severe disease with SARS-Covid-19 infection in a multi-site UK acute Hospital Trust. Medrxiv.

[32] Mahanta S., Chowdhury P., Gogoi N., Goswami N., Borah D., Kumar R., Chetia D., Borah P., Buragohain A.K., Gogoi B. (2020). Potential anti-viral activity of approved repurposed drug against main protease of SARS-CoV-2: An *in silico* based approach [published online ahead of print, 25 May 2020]. J. Biomol. Struct. Dyn..

[33] Vatansever E.C., Yang K., Kratch K.C., Drelich A., Cho C.C., Mellot D.M., Xu S., Tseng C.T.K., Liu W.R. (2020). Targeting the SARS-CoV-2 Main Protease to Repurpose Drugs for COVID-19. BioRxiv.

[34] Tariq A., Mateen R., Sohail A.S., Saleem M. (2020). Paromomycin: A Potential Dual Targeted Drug Effectively Inhibits both Spike (S1) and Main Protease of COVID-19. Int. J. Infect. Dis..

[35] Duarte R.R., Copertino D.C., Iñiguez L.P., Marston J.L., Nixon D.F., Powell T.R. (2020). Repurposing FDA-Approved Drugs for COVID-19 Using a Data-Driven Approach. ChemRxiv.

[36] Kumar V., Jena M. (2020). *In silico* virtual screening-based study of nutraceuticals predicts the therapeutic potentials of folic acid and its derivatives against COVID-19. Res. Sq..

[37] Tsuji M. (2020). Potential anti-SARS-CoV-2 drug candidates identified through virtual screening of the ChEMBL database for compounds that target the main coronavirus protease. FEBS Open Bio.

[38] Durdagi S., Aksoydan B., Dogan B., Sahin K., Shahraki A., Birgül-İyison N. (2020). Screening of Clinically Approved and Investigation Drugs as Potential Inhibitors of SARS-CoV-2 Main Protease and Spike Receptor-Binding Domain Bound with ACE2 COVID19 Target Proteins: A Virtual Drug Repurposing Study. ChemRxiv.

[39] Verma D., Kapoor S., Das S., Thakur K. (2020). Potential Inhibitors of SARS-CoV-2 Main Protease (M^pro^) Identified from the Library of FDA Approved Drugs Using Molecular Docking Studies. Preprints.org..

[40] Kandeel M., Al-Nazawi M. (2020). Virtual screening and repurposing of FDA approved drugs against COVID-19 main protease. Life Sci..

[41] Rosa S.G.V., Santos W.C. (2020). Clinical trials on drug repositioning for COVID-19 treatment. Rev. Panam Salud Publica.

[42] Vafaei S., Razmi M., Mansoori M., Asadi-Lari M., Madjd Z. (2019). Spotlight of Remdesivir in Comparison with Ribavirin, Favipiravir, Oseltamivir and Umifenovir in Coronavirus Disease 2019 (COVID-19) Pandemic. Favipiravir Oseltamivir Umifenovir Coronavirus Dis..

[43] Veljkovic V., Cosic I., Dimitrijevic B. (1985). Is it possible to analyze DNA and protein sequences by the methods of digital signal processing?. IEEE Trans. Biomed. Eng..

[44] Veljkovic V., Slavic I. (1972). Simple general-model pseudopotential. Phys. Rev. Lett..

[45] Stewart J.J., MOPAC2016 (2016). Stewart Computational Chemistry.

[46] Stewart J.J.P. (2013). Optimization of parameters for semiempirical methods VI: More modifications to the NDDO approximations and re-optimization of parameters. J. Mol. Model..

[47] Trott O., Olson A.J. (2010). AutoDock Vina: Improving the speed and accuracy of docking with a new scoring function, efficient optimization and multithreading. J. Comput. Chem..

[48] Dallakyan S., Olson A.J. (2015). Small-Molecule Library Screening by Docking with PyRx. Methods Mol. Biol..

[49] Biovia D.S. (2016). Discovery Studio Modeling Environment (Release 2017).

[50] Maestro V. (2016). Schrödinger Release 2017-1: Maestro.

[51] (2012). Origin.

