# Supplementary material for: Drug Repurposing for Candidate SARS-CoV-2 Main Protease Inhibitors by a Novel In Silico Method
Source: Molecules. 2020 Aug 23;25(17):3830. doi: 10.3390/molecules25173830 (PMC7503980; doi:10.3390/molecules25173830)
Supplement: Supplementary file 1 [file molecules-25-03830-s001.zip › molecules-884035-supplementary-revised - original/Supplementary Files/S1_Table.pdf]

From	To	Type of interaction	
SER1	GLU166	Hydrogen Bond; Electrostatic	Salt Bridge
ARG4	GLU290	Hydrogen Bond; Electrostatic	Salt Bridge
SER1	GLU166	Hydrogen Bond; Electrostatic	Salt Bridge
ARG4	GLU290	Hydrogen Bond; Electrostatic	Salt Bridge
ARG4	GLU290	Electrostatic	Attractive Charge
ARG4	GLU290	Electrostatic	Attractive Charge
SER1	PHE140	Hydrogen Bond	Conventional Hydrogen Bond
ARG4	LYS137	Hydrogen Bond	Conventional Hydrogen Bond
ALA7	VAL125	Hydrogen Bond	Conventional Hydrogen Bond
SER10	SER10	Hydrogen Bond	Conventional Hydrogen Bond
GLY11	GLU14	Hydrogen Bond	Conventional Hydrogen Bond
VAL125	ALA7	Hydrogen Bond	Conventional Hydrogen Bond
SER139	GLN299	Hydrogen Bond	Conventional Hydrogen Bond
PHE140	SER1	Hydrogen Bond	Conventional Hydrogen Bond
ARG298	SER123	Hydrogen Bond	Conventional Hydrogen Bond
SER1	PHE140	Hydrogen Bond	Conventional Hydrogen Bond
ARG4	LYS137	Hydrogen Bond	Conventional Hydrogen Bond
ALA7	VAL125	Hydrogen Bond	Conventional Hydrogen Bond
SER10	SER10	Hydrogen Bond	Conventional Hydrogen Bond
GLY11	GLU14	Hydrogen Bond	Conventional Hydrogen Bond
VAL125	ALA7	Hydrogen Bond	Conventional Hydrogen Bond
SER139	GLN299	Hydrogen Bond	Conventional Hydrogen Bond
PHE140	SER1	Hydrogen Bond	Conventional Hydrogen Bond
ARG298	SER123	Hydrogen Bond	Conventional Hydrogen Bond
GLY2	GLY138	Hydrogen Bond	Carbon Hydrogen Bond
GLY2	GLY138	Hydrogen Bond	Carbon Hydrogen Bond
ALA7	VAL125	Hydrophobic	Alkyl
PRO9	PRO122	Hydrophobic	Alkyl
PRO122	PRO9	Hydrophobic	Alkyl
VAL125	VAL125	Hydrophobic	Alkyl
ALA285	ALA285	Hydrophobic	Alkyl

ALA285	LEU286	Hydrophobic	Alkyl
ALA7	VAL125	Hydrophobic	Alkyl
ALA285	LEU286	Hydrophobic	Alkyl
TYR126	ARG4	Hydrophobic	Pi-Alkyl
TYR126	MET6	Hydrophobic	Pi-Alkyl
TYR126	ARG4	Hydrophobic	Pi-Alkyl
TYR126	MET6	Hydrophobic	Pi-Alkyl
