# Supplementary figures and images for: Drug Repurposing for Candidate SARS-CoV-2 Main Protease Inhibitors by a Novel In Silico Method
Source: Molecules. 2020 Aug 23;25(17):3830. doi: 10.3390/molecules25173830 (PMC7503980; doi:10.3390/molecules25173830)

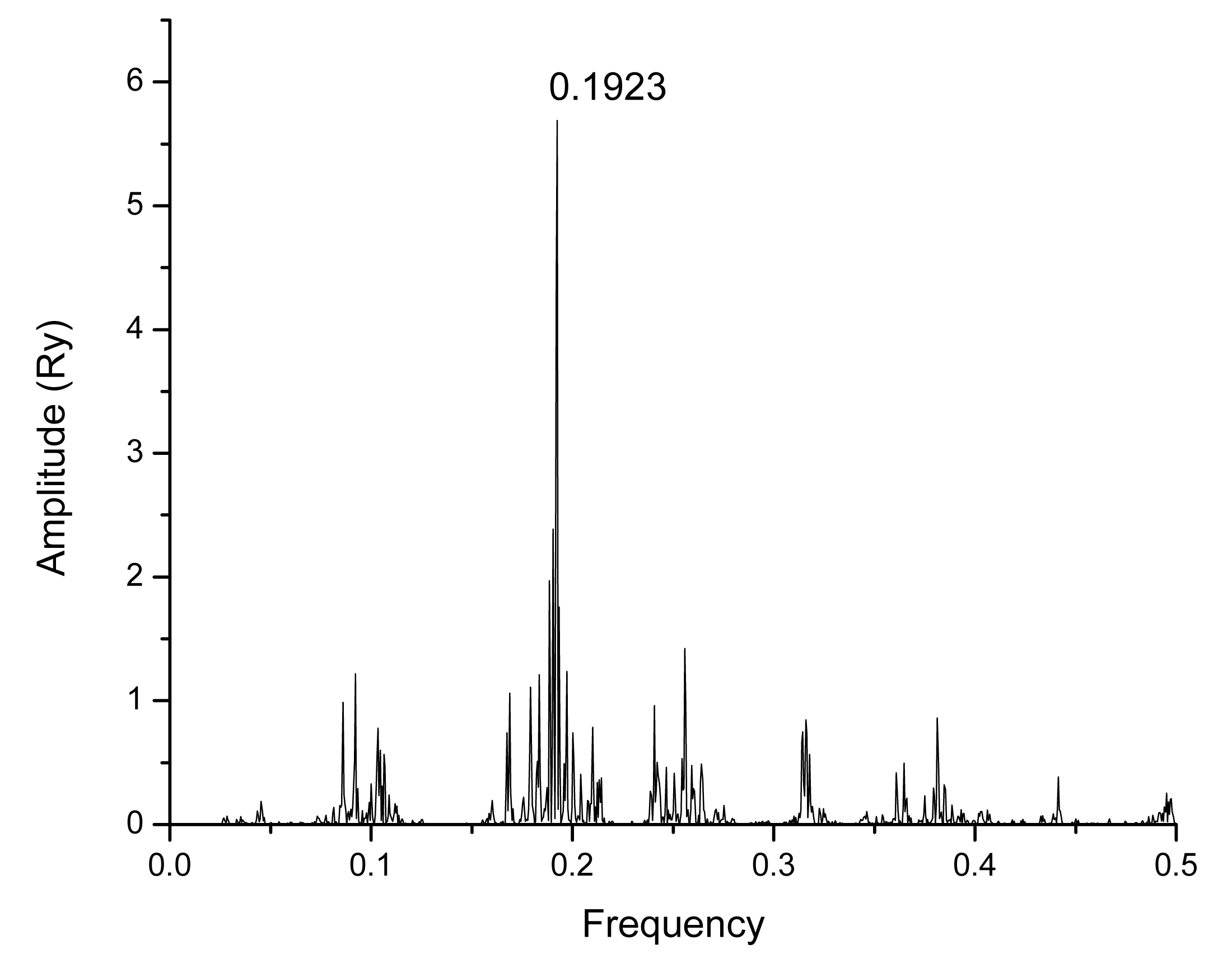

Supplement: Supplementary file 1 [file molecules-25-03830-s001.zip › molecules-884035-supplementary-revised - original/Supplementary Files/S10_Fig.tif]

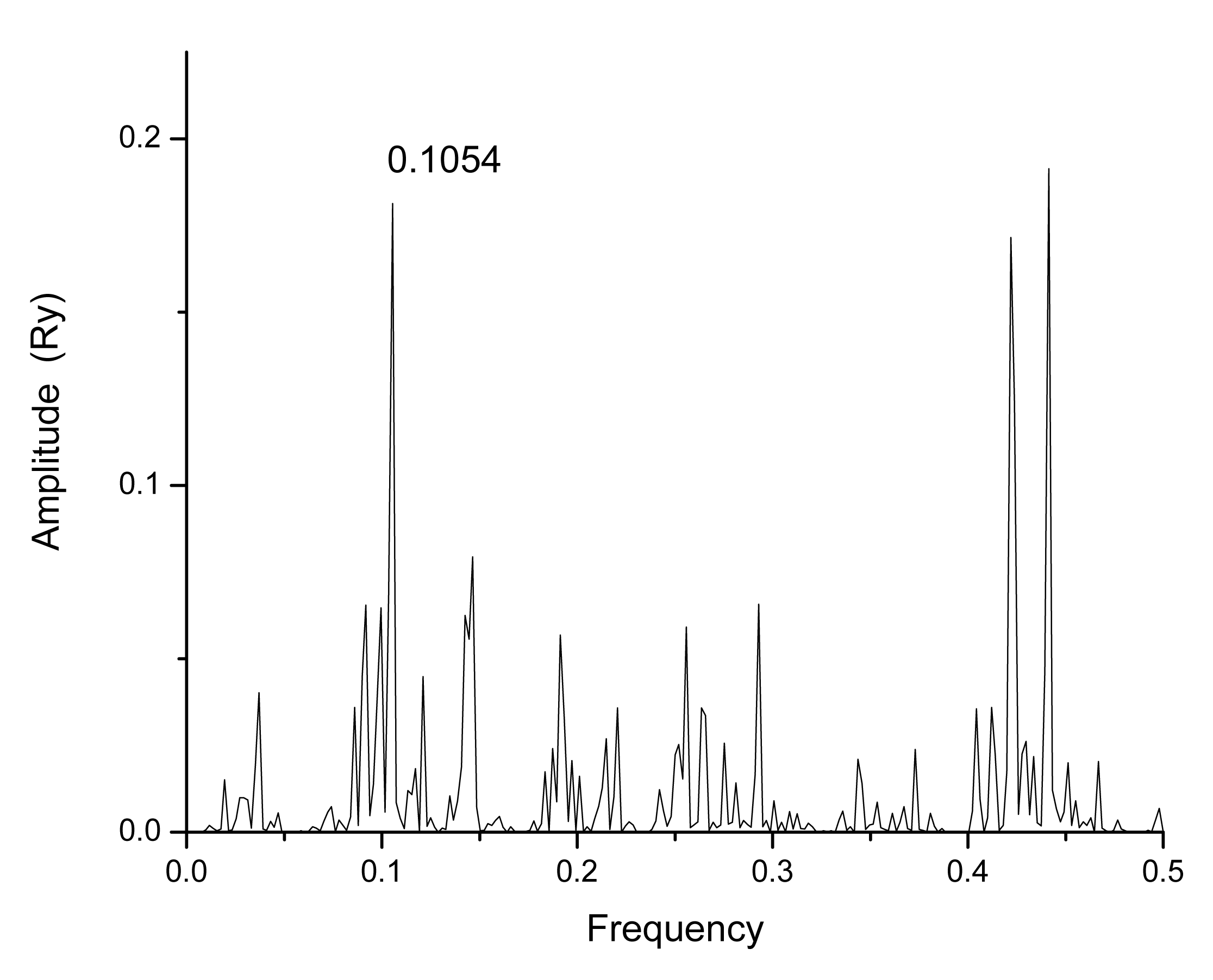

Supplement: Supplementary file 1 [file molecules-25-03830-s001.zip › molecules-884035-supplementary-revised - original/Supplementary Files/S11_Fig.tif]

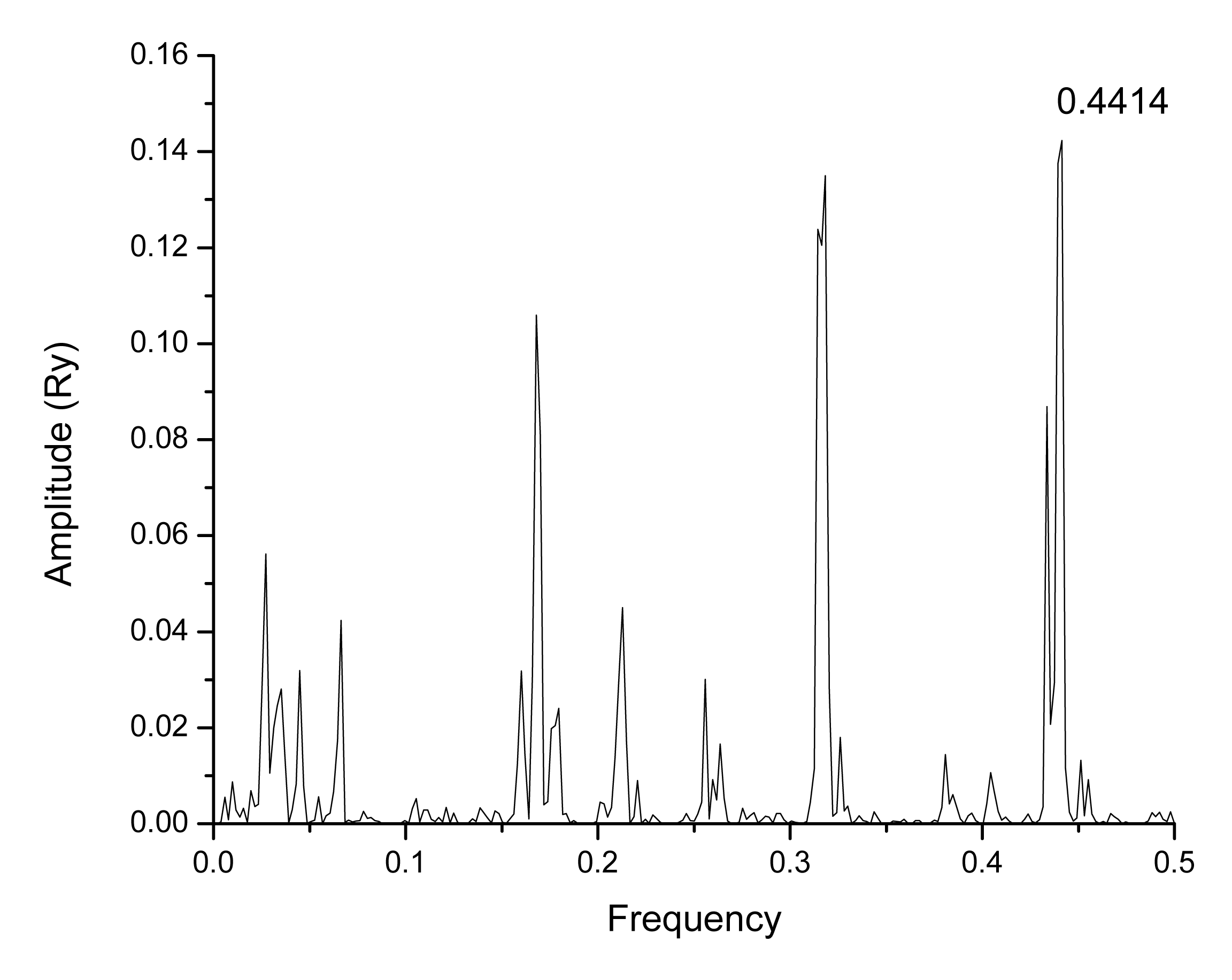

Supplement: Supplementary file 1 [file molecules-25-03830-s001.zip › molecules-884035-supplementary-revised - original/Supplementary Files/S12_Fig.tif]

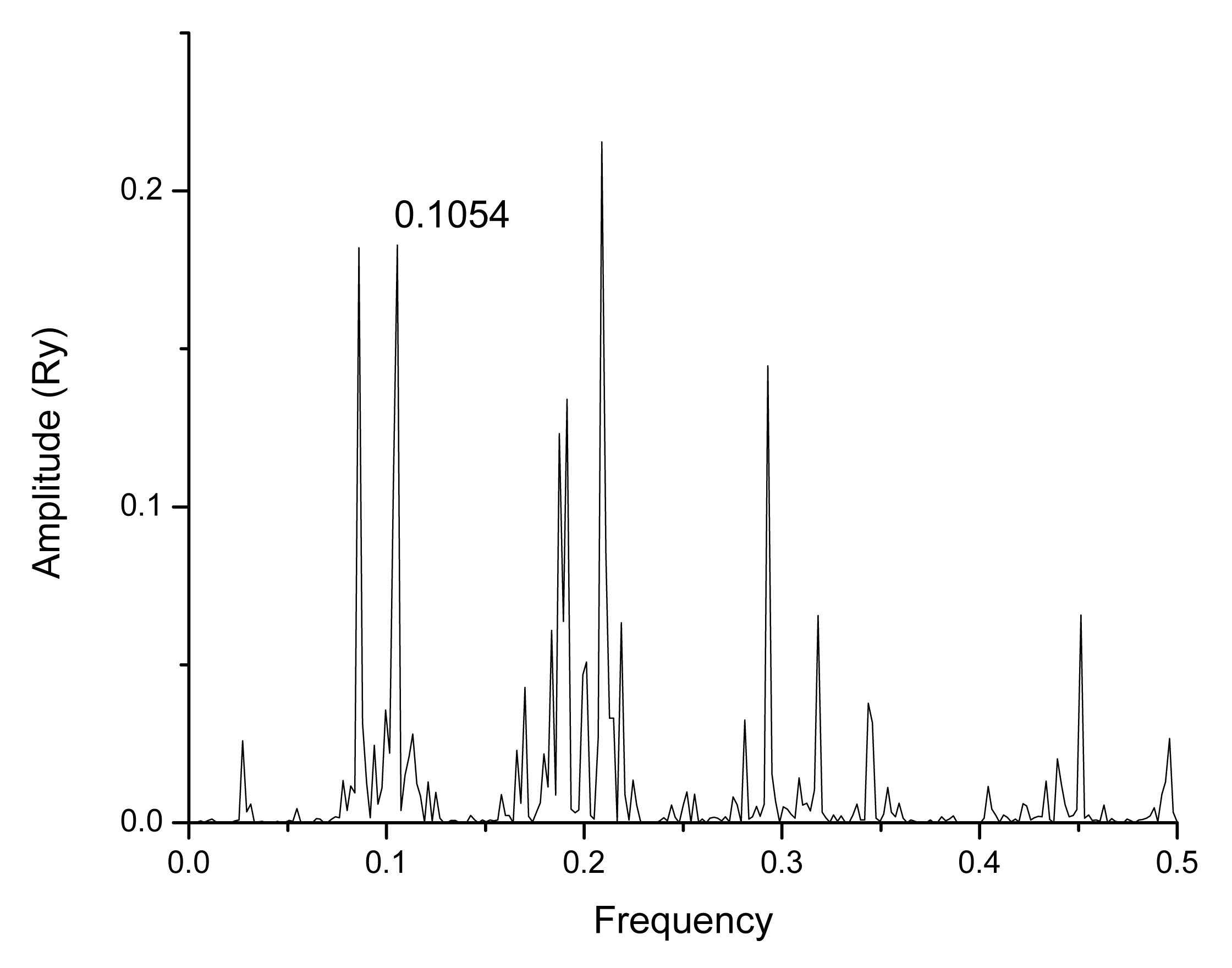

Supplement: Supplementary file 1 [file molecules-25-03830-s001.zip › molecules-884035-supplementary-revised - original/Supplementary Files/S13_Fig.tif]

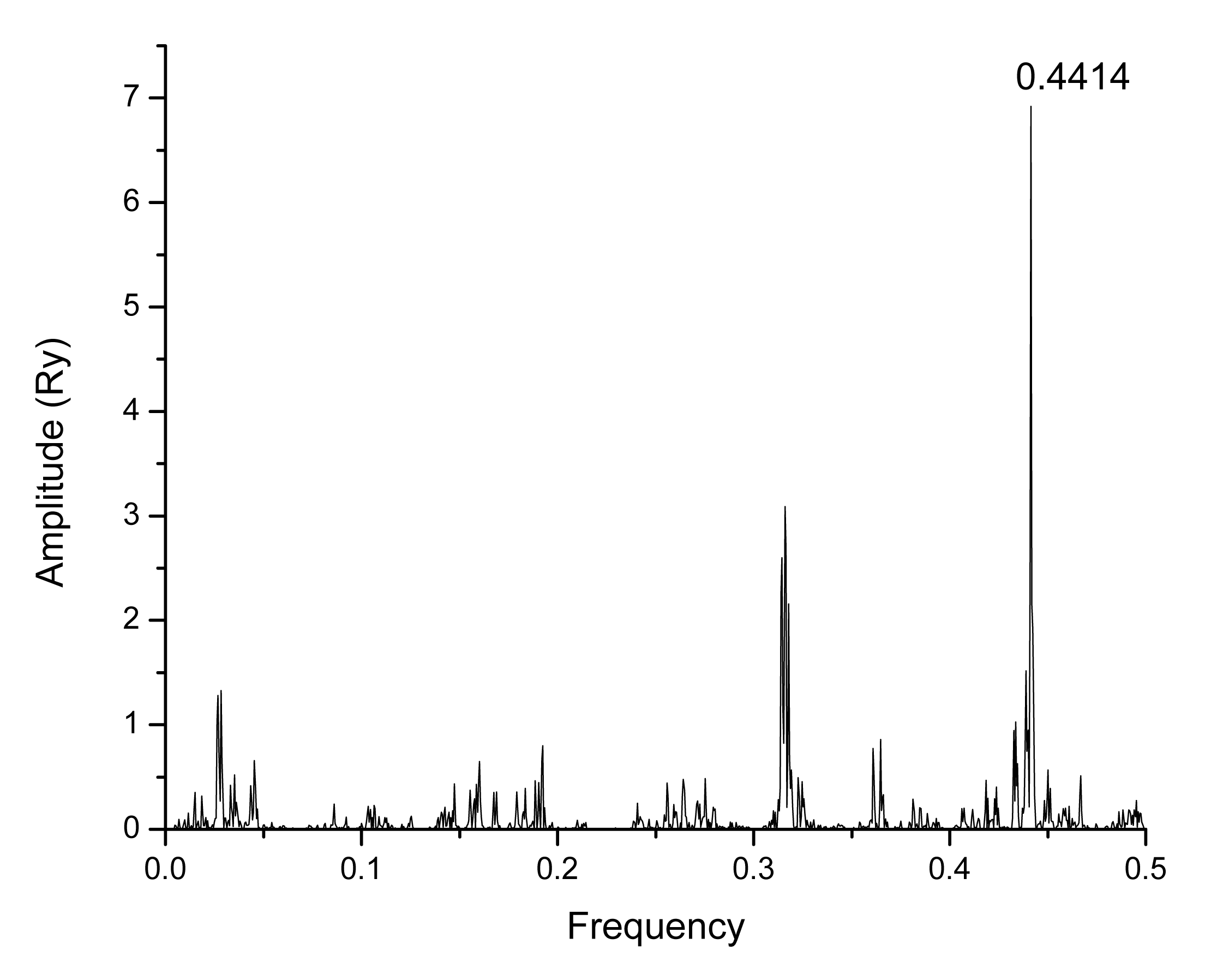

Supplement: Supplementary file 1 [file molecules-25-03830-s001.zip › molecules-884035-supplementary-revised - original/Supplementary Files/S14_Fig.tif]

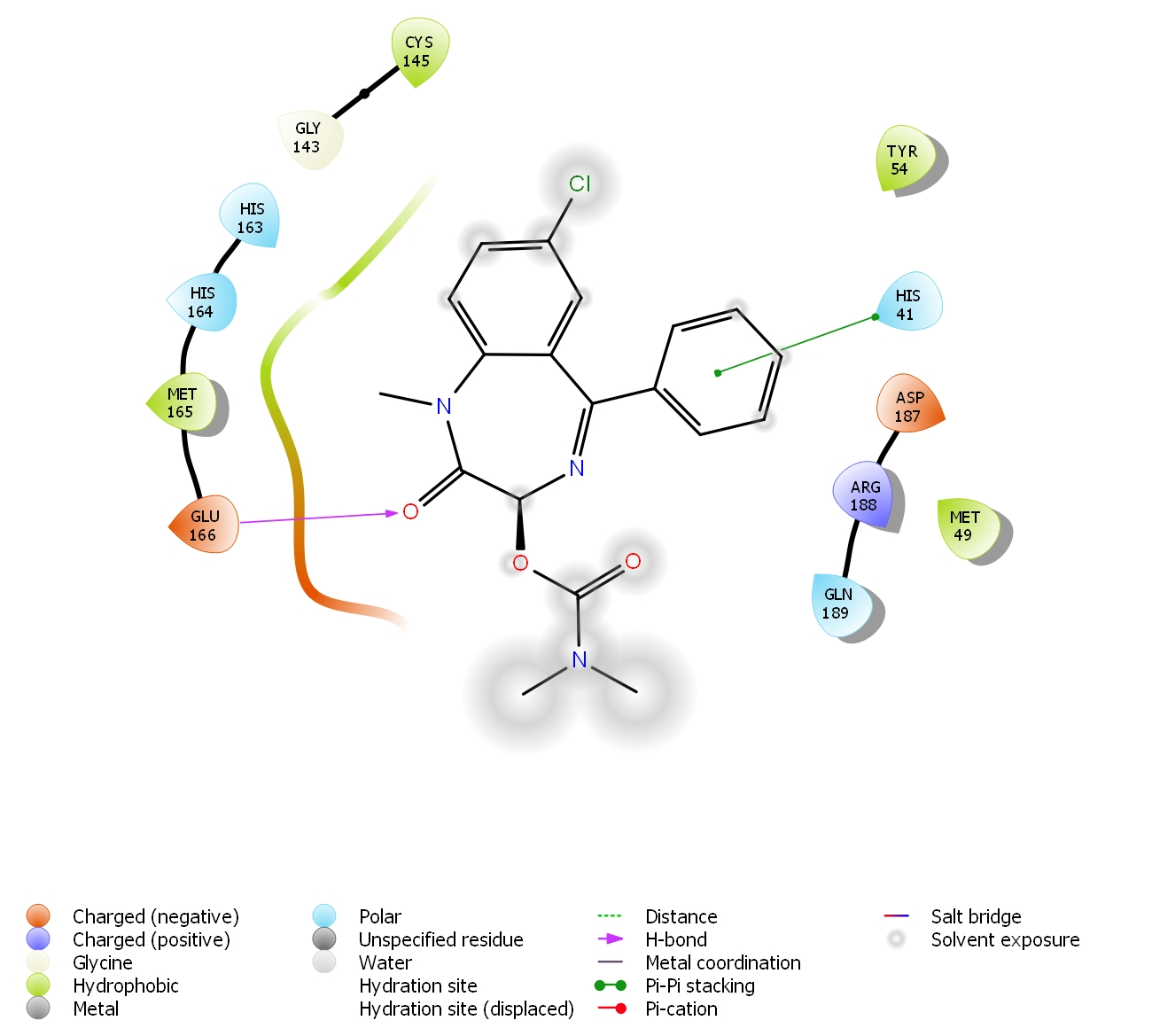

Supplement: Supplementary file 1 [file molecules-25-03830-s001.zip › molecules-884035-supplementary-revised - original/Supplementary Files/S1_Fig.tiff]

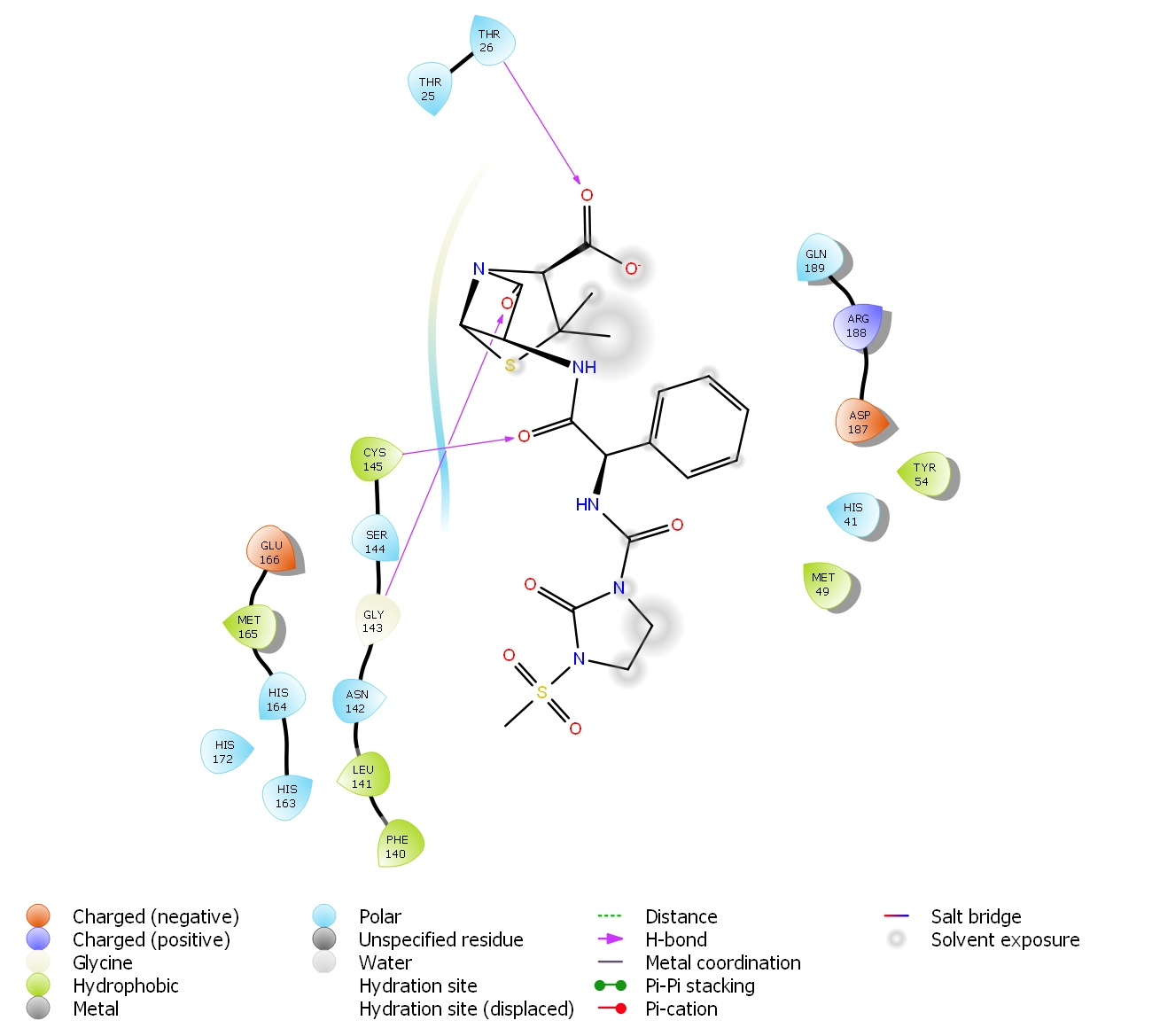

Supplement: Supplementary file 1 [file molecules-25-03830-s001.zip › molecules-884035-supplementary-revised - original/Supplementary Files/S2_Fig.tiff]

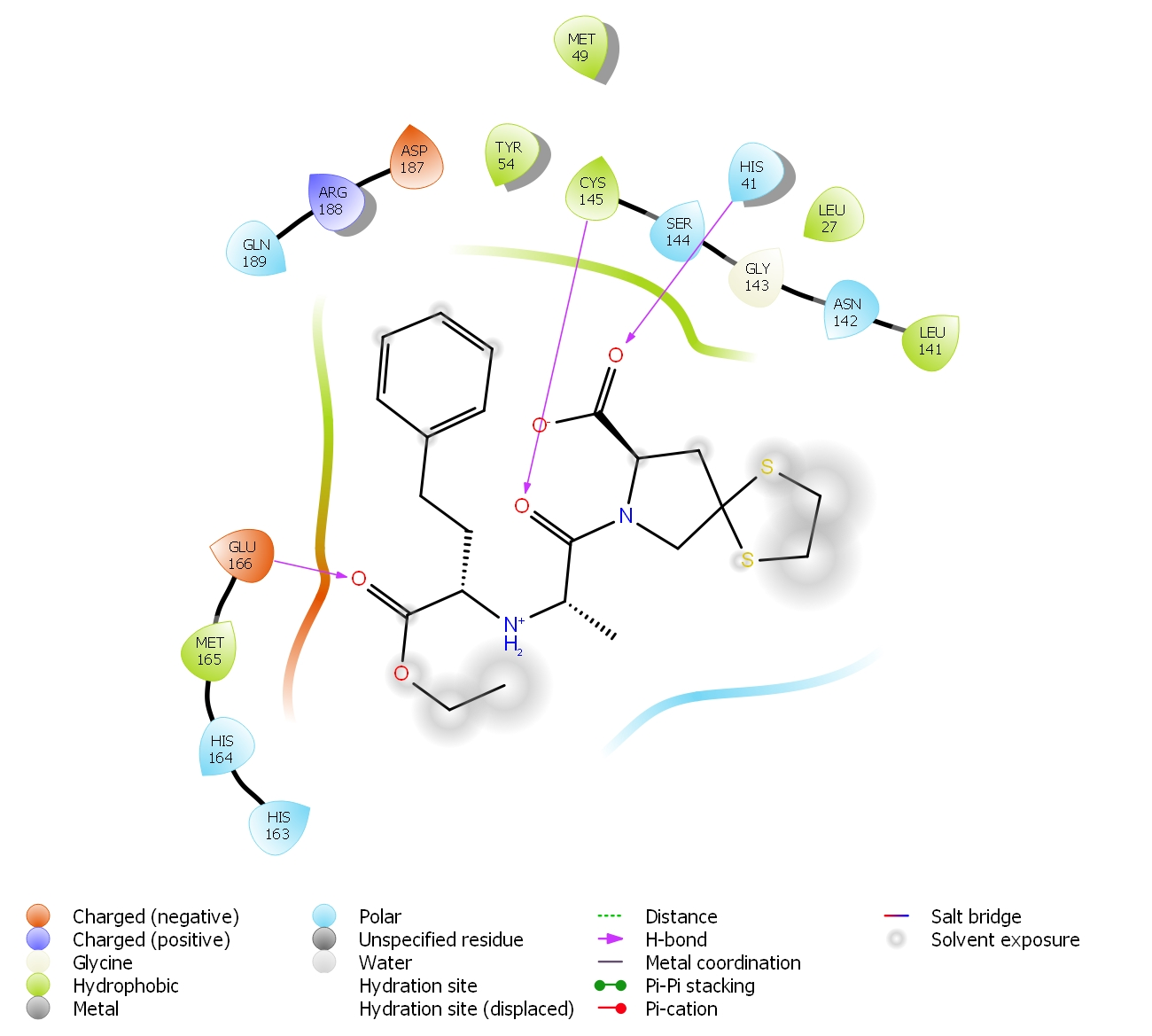

Supplement: Supplementary file 1 [file molecules-25-03830-s001.zip › molecules-884035-supplementary-revised - original/Supplementary Files/S3_Fig.tiff]

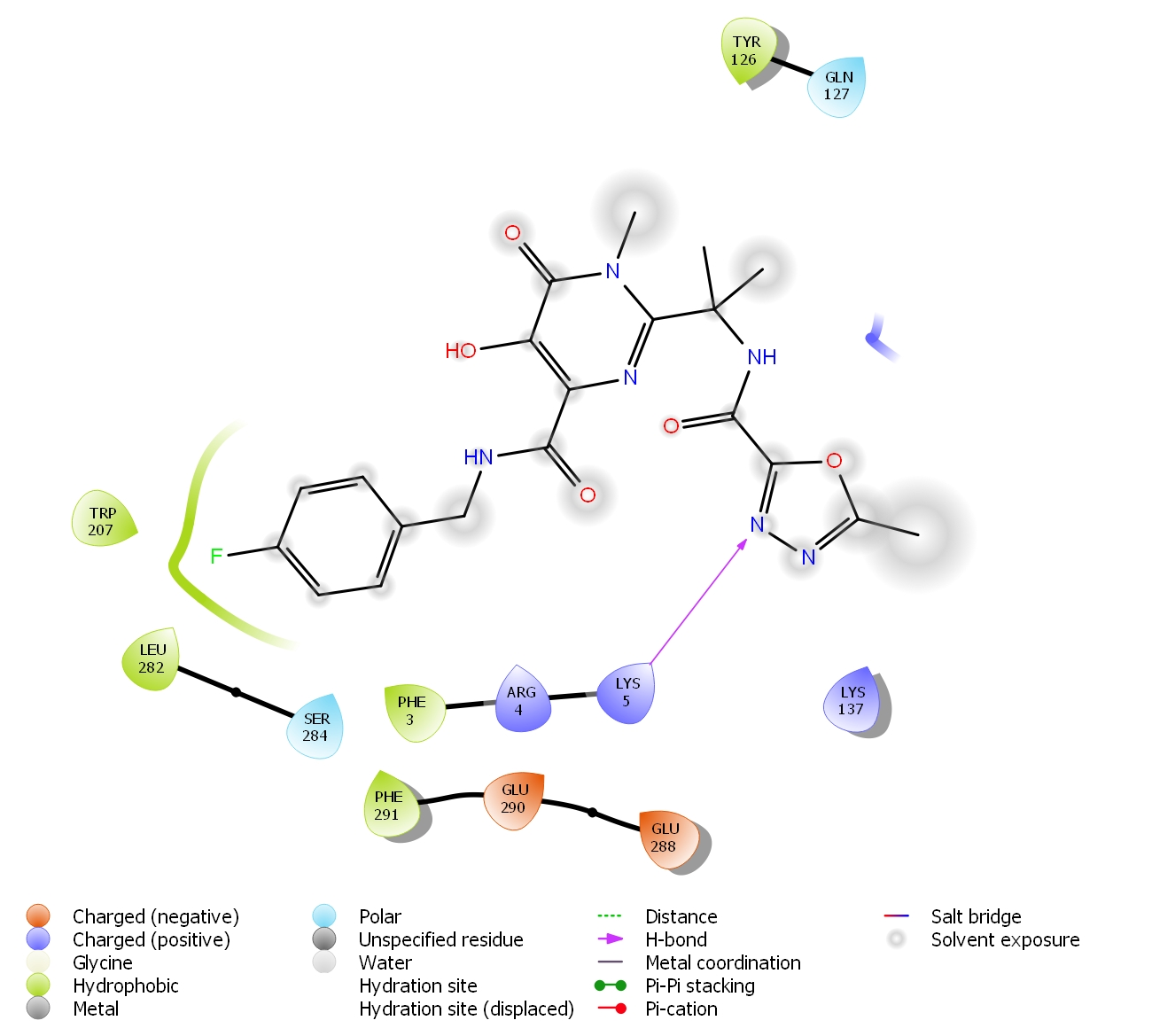

Supplement: Supplementary file 1 [file molecules-25-03830-s001.zip › molecules-884035-supplementary-revised - original/Supplementary Files/S4_Fig.tiff]

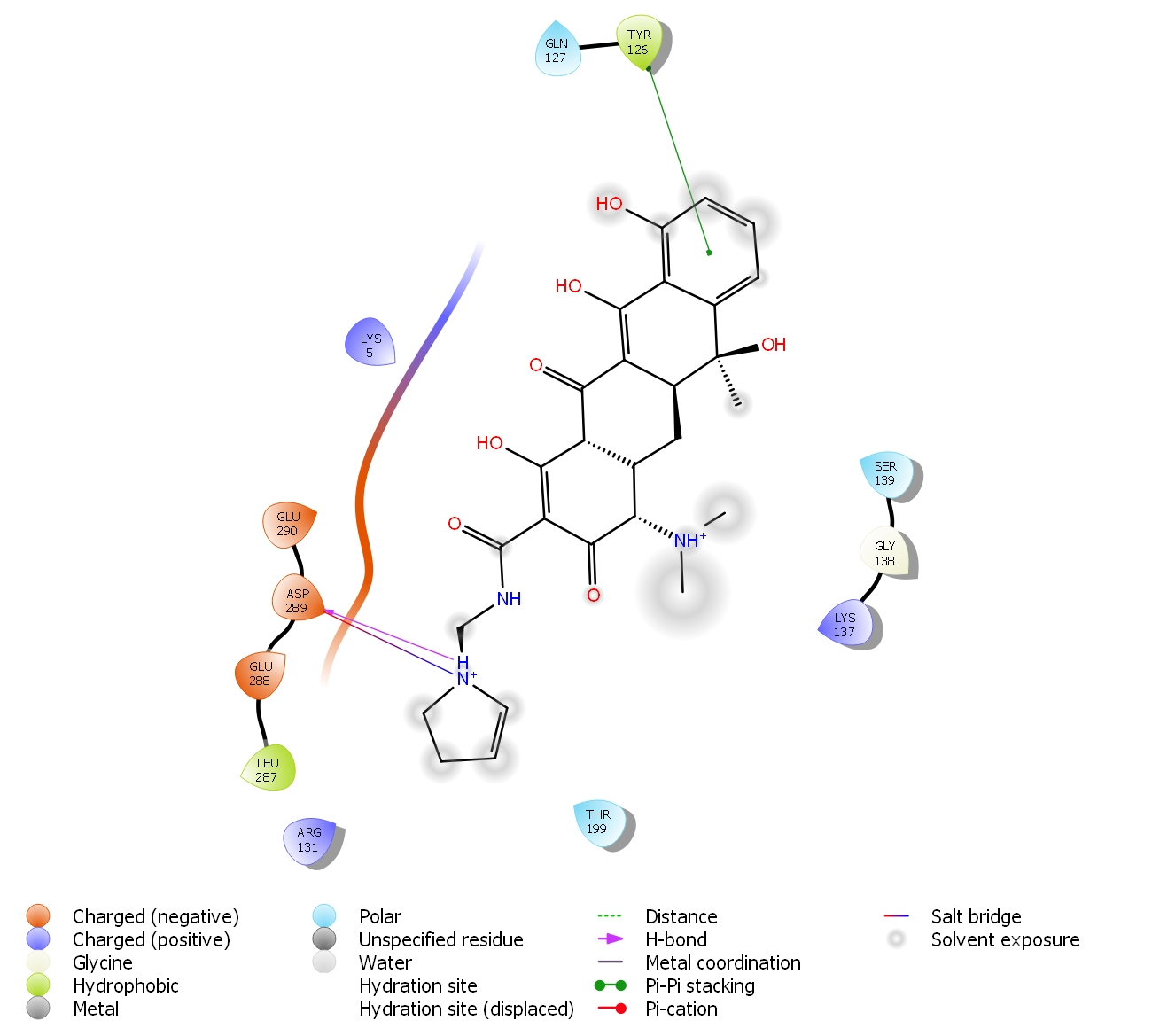

Supplement: Supplementary file 1 [file molecules-25-03830-s001.zip › molecules-884035-supplementary-revised - original/Supplementary Files/S5_Fig.tiff]

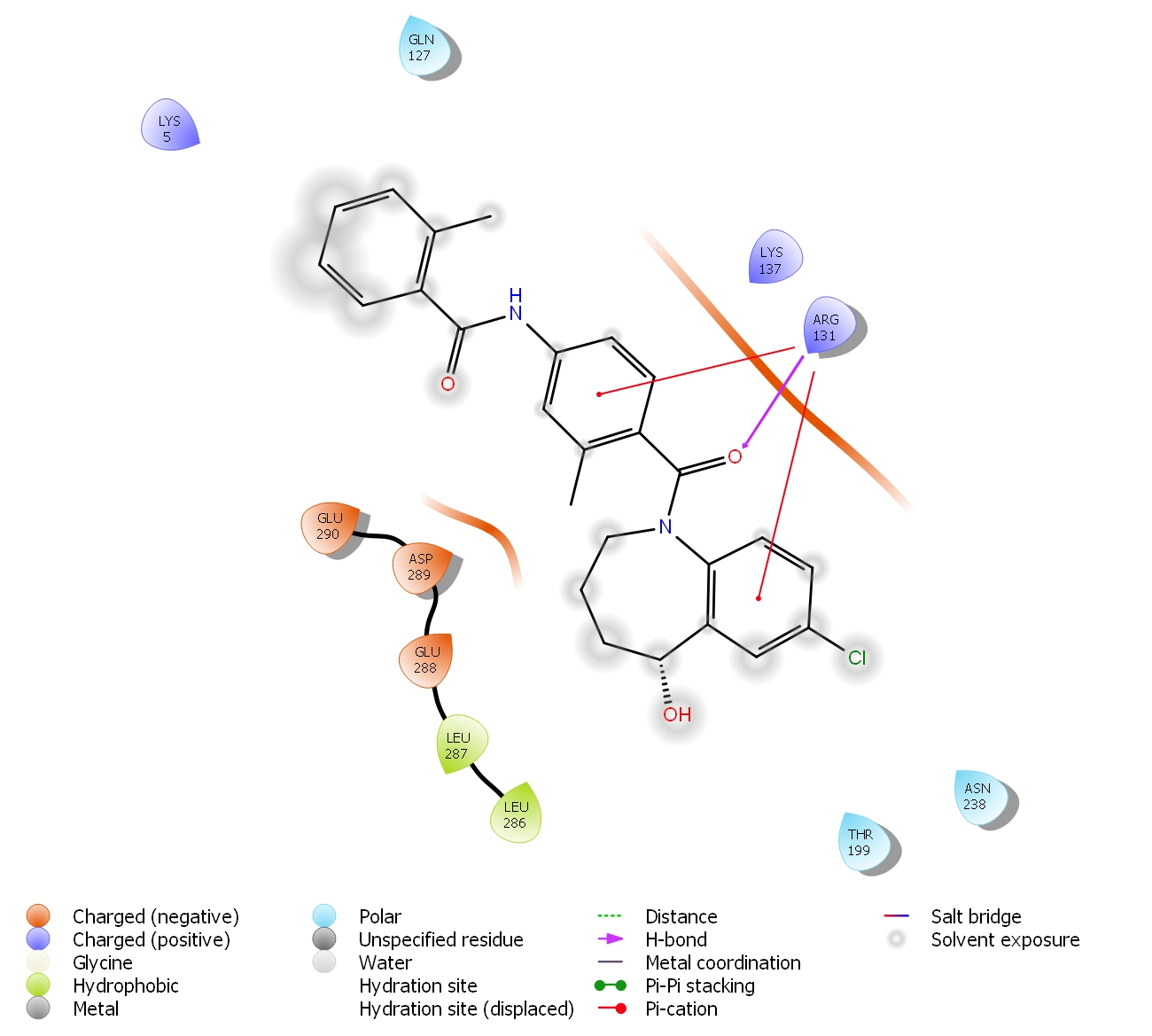

Supplement: Supplementary file 1 [file molecules-25-03830-s001.zip › molecules-884035-supplementary-revised - original/Supplementary Files/S6_Fig.tiff]

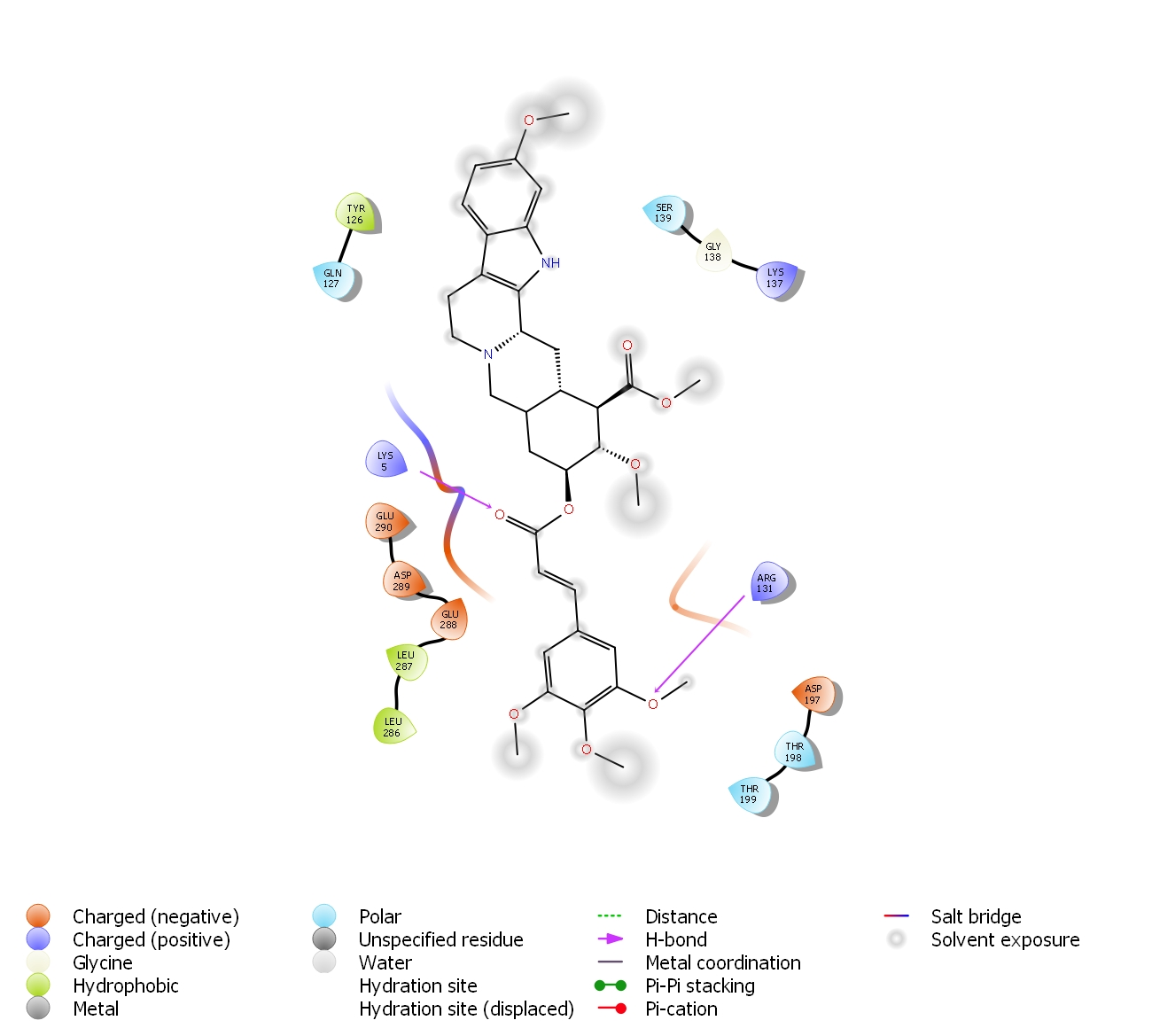

Supplement: Supplementary file 1 [file molecules-25-03830-s001.zip › molecules-884035-supplementary-revised - original/Supplementary Files/S7_Fig.tiff]

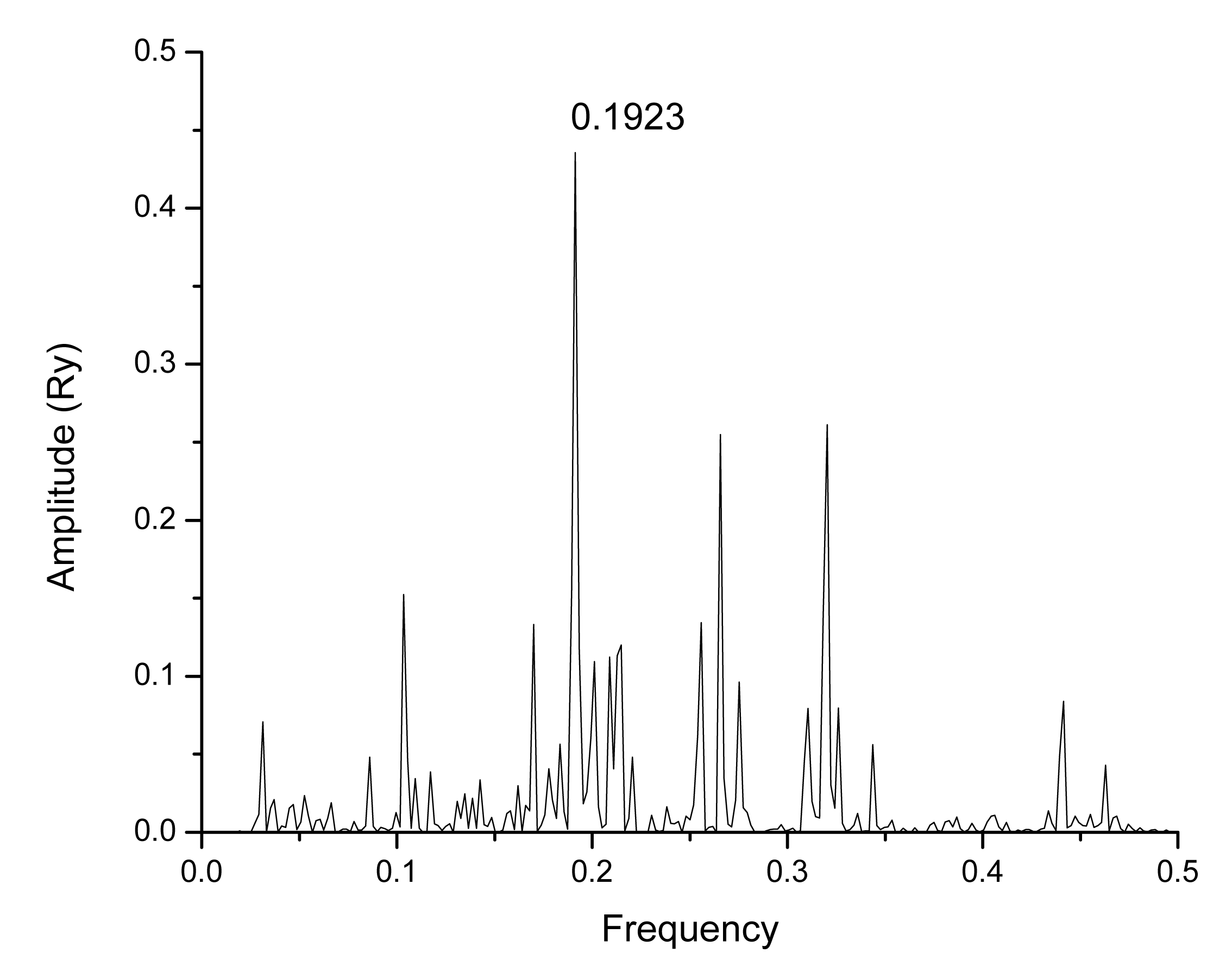

Supplement: Supplementary file 1 [file molecules-25-03830-s001.zip › molecules-884035-supplementary-revised - original/Supplementary Files/S9_Fig.tif]
